# HIV-1 RNAs are Not Part of the Argonaute 2 Associated RNA Interference Pathway in Macrophages

**DOI:** 10.1371/journal.pone.0132127

**Published:** 2015-07-30

**Authors:** Valentina Vongrad, Jochen Imig, Pejman Mohammadi, Shivendra Kishore, Lukasz Jaskiewicz, Jonathan Hall, Huldrych F. Günthard, Niko Beerenwinkel, Karin J. Metzner

**Affiliations:** 1 University Hospital Zurich, Division of Infectious Diseases and Hospital Epidemiology, University of Zurich, Zurich, Switzerland; 2 Institute of Medical Virology, University of Zurich, Zurich, Switzerland; 3 ETH Zurich, Institute of Pharmaceutical Sciences, Zurich, Switzerland; 4 ETH Zurich, Department of Biosystems Science and Engineering, Basel, Switzerland; 5 SIB Swiss Institute of Bioinformatics, Basel, Switzerland; 6 University of Basel, Computational and Systems Biology, Basel, Switzerland; French National Center for Scientific Research - Institut de biologie moléculaire et cellulaire, FRANCE

## Abstract

**Background:**

MiRNAs and other small noncoding RNAs (sncRNAs) are key players in post-transcriptional gene regulation. HIV-1 derived small noncoding RNAs (sncRNAs) have been described in HIV-1 infected cells, but their biological functions still remain to be elucidated. Here, we approached the question whether viral sncRNAs may play a role in the RNA interference (RNAi) pathway or whether viral mRNAs are targeted by cellular miRNAs in human monocyte derived macrophages (MDM).

**Methods:**

The incorporation of viral sncRNAs and/or their target RNAs into RNA-induced silencing complex was investigated using photoactivatable ribonucleoside-induced cross-linking and immunoprecipitation (PAR-CLIP) as well as high-throughput sequencing of RNA isolated by cross-linking immunoprecipitation (HITS-CLIP), which capture Argonaute2-bound miRNAs and their target RNAs. HIV-1 infected monocyte-derived macrophages (MDM) were chosen as target cells, as they have previously been shown to express HIV-1 sncRNAs. In addition, we applied small RNA deep sequencing to study differential cellular miRNA expression in HIV-1 infected versus non-infected MDMs.

**Results and Conclusion:**

PAR-CLIP and HITS-CLIP data demonstrated the absence of HIV-1 RNAs in Ago2-RISC, although the presence of a multitude of HIV-1 sncRNAs in HIV-1 infected MDMs was confirmed by small RNA sequencing. Small RNA sequencing revealed that 1.4% of all sncRNAs were of HIV-1 origin. However, neither HIV-1 derived sncRNAs nor putative HIV-1 target sequences incorporated into Ago2-RISC were identified suggesting that HIV-1 sncRNAs are not involved in the canonical RNAi pathway nor is HIV-1 targeted by this pathway in HIV-1 infected macrophages.

## Introduction

MicroRNAs (miRNAs) are a class of short RNA molecules of 19–24 nucleotide length that participate in the RNA interference (RNAi) pathway to negatively regulate target genes at the post-transcriptional level [1]. The so-called “guide strand” is incorporated into the RNA-induced silencing complex (RISC), while the “passenger strand” is mostly degraded. The guide strand fulfills its function by recognizing its target RNA through a 7-mer (position 2–8 at the 5’ end of the miRNA) complementary to the respective target sequence [2].

Numerous studies have shown that not only the genomes of multicellular organisms encode miRNAs, but also viral genomes [3–7]. Viral miRNAs have beneficial functions for the virus, for instance, inhibiting immune responses or establishing a latent reservoir [8–14]. Therefore, the identification and characterization of viral and cellular miRNAs is of importance for the understanding of virus-host interactions, virus replication, latency, and eventually for the development of novel antiviral therapies [15–17].

The first virus-derived miRNAs were identified in Epstein-Barr virus-infected human B cells [3]. Soon after, miRNAs were shown to be encoded in diverse virus genera, most of them in DNA viruses [18]. The existence of miRNAs derived from RNA viruses, however, has been a source of controversy. For example, the presence of virus-derived miRNAs would lead to a potential degradation of the virus’ own genome. Furthermore, as most RNA viruses replicate in the cytoplasm outside of the nucleus, they should be inaccessible to Drosha which is required for the biogenesis of functionally active miRNAs [19]. Later it was shown that the retrovirus Bovine Leukemia Virus expresses viral miRNAs without degradation of its own genome by using RNA Polymerase III for their transcription, hence evading the recognition of the pri-miRNA transcripts by Drosha in the nucleus [6]. This finding suggests that viral adaptation is possible by bypassing canonical RNAi pathways.

We and others have identified sncRNAs encoded by HIV-1. Omoto *et al*. found a sncRNA in *nef*, called miR-N367 [13,20]. One year later, a HIV-1-derived siRNA located in *env* was identified. Overexpression assays showed that downregulated *env* mRNA subsequently suppressed HIV-1 replication [21]. Further miRNAs derived from the long terminal repeat (LTR) were described [12,22,23], namely miR-TAR-5p and miR-TAR-3p, which were suggested to play a role in chromatin remodeling and apoptosis [12,22]. Kaul *et al*. identified and characterized miR-H1 located in the LTR [24,25], which may have the potential to regulate the apoptosis-antagonizing transcription factor (AATF). Applying large-scale analysis of small RNAs from HIV-1 infected cells revealed the expression of numerous sncRNAs encoded by HIV-1 [26–29]. They were shown to be distributed all throughout the viral genome, and some were expressed in antisense orientation [27,28]. Contradictory to these reports, two studies failed to identify HIV-1 sncRNAs in HIV-1 infected cell lines. Although Pfeffer *et al*. detected two sncRNAs in an HIV-1 infected T cell line, they declared them as degradation products, because these RNAs had not been predicted to form stable hairpin structures [7]. Lin and Cullen also did not detect HIV-1 sncRNAs in two different T cell lines by Northern Blot and cDNA cloning [30]. Recently, Whisnant *et al*. showed that HIV-1 sncRNAs were not associated with RISC in infected cell lines [29].

We have previously shown that HIV-1 sncRNAs were expressed in monocyte-derived macrophages (MDM) [28]. Macrophages are important target cells for HIV-1 *in vivo* and contribute to the long-lasting viral latent reservoir [31]. In the present study, we addressed two central questions. First, are HIV-1 sncRNAs incorporated in RISC and actively involved in the RNAi pathway in MDMs? And second, is the HIV-1 RNA targeted by cellular miRNAs in MDMs?

## Materials and Methods

### Ethics statement

Monocytes used for this study were derived from buffy coats obtained from healthy blood donors, as anonymously provided by the Blood Donation Service Zurich, Swiss Red Cross, Schlieren, Switzerland. Written consent for the use of buffy coats not required for medical treatment for research purposes was obtained from blood donors by the Blood Donation Centre.

### Virus stock and cell culture

HIV-1_JR-FL_ virus stock was generated by transfection of 293T cells with the HIV-1 full-length plasmid pJR-FL. Virus stock was harvested 48 hours post transfection and filtered through a 0.45 μm pore-size filter. The tissue culture infectious dose 50 (TCID_50_) was estimated as described elsewhere [32]. All reads were aligned to HIV-1_JR-FL_ (GenBank: U63632.1) and LTR from HIV-1_JRCSF_ (GenBank: M38429.1).

Primary human monocytes from HIV-1 negative blood donors were isolated with anti-CD14 coated magnetic beads (Miltenyi Biotech) from buffy coats and cultured one week in RPMI supplemented with 1% penicillin/streptomycin, 10% human serum (Sigma) and 0.02 μg/ml M-CSF (macrophage colony stimulating factor). Seven days after isolation the macrophages were washed in PBS supplemented with 2% FCS to remove not adherent and dead cells and the medium was replaced and cultured in RPMI supplemented with 1% penicillin/streptomycin and 10% FCS. After another week, the differentiated macrophages were infected with HIV-1_JR-FL_ with a multiplicity of infection (MOI) of 0.1. The cells were washed the next day with PBS supplemented with 2% FCS. The culture media was exchanged every 3 to 4 days [28]. Cells were analyzed by microscopy HIV-1 replication was monitored by p24 ELISA as described (adapted from [33]).

### Ago2 immunoprecipitation

The Ago2-IPs were performed under conditions described in [34]. The PAR-CLIP Ago2-autoradiogram is shown in Fig A in S1 File. The efficiency of the Ago2-IPs was quantified by qPCR for cellular miRNAs (miR-21 and miR-23a) and compared to control RNAs (5S rRNA) from Ago2-IP (Fig B in S1 File). Control IPs were performed with a non-specific rat serum IgG (Sigma).

### Ago2 photoactivatable ribonucleoside-induced cross-linking and immunoprecipitation (PAR-CLIP)

The total number of 30 to 60 million MDMs per donor were infected with HIV-1_JR-FL_. Two weeks post infection, 4-thiouridine (Sigma) was added to the cell culture at a final concentration of 100 μM for 16 hours. The cells were washed with ice-cold PBS before cross- linking twice with 150 mJ/cm^2^ at 365 nm on ice. Subsequently, the cells were lysed in NP40 lysis buffer (50 mM HEPES, pH 7.5, 150 mM KCl, 0.5% IGEPAL, 0.5 mM DTT, 2 mM EDTA, complete protease inhibitor EDTA-free (Roche), 50 U/mL RNasin, Promega) and snap frozen. The PAR-CLIP assay was processed as previously described [35,36]. Briefly, the cleared cell lysates were treated with RNase T1 (NEB) at a final concentration of 5 U/ml for 15 min at RT. Ago2 was immunoprecipitated with Ago2-antibody clone 11A9 (Sigma) coated ProtG magnetic beads (Life Technologies). Purified Ago2 protein was treated with RNase T1 at 20 U/μl for 15 min at RT. Subsequently, the beads were washed and dephosphorylated with Calf intestinal alkaline phosphatase (NEB, 1U/μL) for 30 min at 37°C in dephosphorylation buffer (NEB buffer 3). After washing, the beads were incubated for 30 min at 37°C with 200 U/mL T4 polynucleotide kinase, 3’-phosphatase free (Roche), and radioactive 10 μL ATP ([γ-32P]- 6000 Ci/mmol 10 mCi/mL, final volume 100 μL, Perkin-Elmer) to radioactive label the cross-linked RNA. The protein-RNA complexes were eluted with 100 μL 1x LDS loading buffer (Life Technologies) at 95°C, separated by SDS-PAGE (NuPageNovex 4–12% Bis-Tris Midi Gel) in MOPS buffer and transferred onto a nitrocellulose membrane. The membrane was washed for 5 min in PBS, exposed to a phosphoimager screen, and 120 kDa bands were excised. The membranes were digested with 36 μg/μl (+/- 8 μg/μl) proteinase K (PCR grade, Roche) in 1x proteinase K digestion buffer (50 mM Tris/Cl, pH 7.5, 75 mM NaCl, 6.25 mM EDTA, 1% SDS (v/w)) for 15 min at 65°C under agitation. The RNA was recovered by acidic phenol/chloroform extraction, ethanol precipitation, and addition of Glycoblue (Ambion). Thereafter, preadenylated 3’-adapter (IDT DNA Technologies, 5’-TGGAATTCTCGGGTGCCAAGG-3’) was ligated with truncated T4 RNA Ligase 2 (1–249, K227Q, final concentration 2000 U/μL, NEB) overnight on ice. After a denaturing 15% PAA gel separation, RNA ranging from 35 to 70 nt was cut out of gel and extracted in 0.4 M NaCl overnight. Again, the RNA was recovered as described above followed by 5’-RNA Adapter (5’-GUUCAGAGUUCUACAGUCCGACGAUC-3’) ligation using T4 RNA ligase (Fermentas) at 37°C for 1 h. RNA was again gel-purified as described above. RNA was reverse transcribed into cDNA with SuperScript III reverse transcriptase (Invitrogen) according to the manufacturer’s description. Library amplification step was performed with minimal number of PCR cycles just enabling analysis of amplicons via 2.5% agarose gel as determined by a preceding PCR using different PCR cycle numbers. The range of final PCR cycle numbers was between 21 and 24, with the mean of 22.3 and 23.3 for non-infected and HIV-1_JR-FL_ infected samples. PCR was performed using Taq DNA-Polymerase (Sigma-Aldrich as described) with Index primers described in IlluminaTruSeq Small RNA Sample Prep Kit protocol. Amplicons were extracted using QiaExII (Qiagen) according to the purchaser’s protocol. The recovered DNA was sequenced using single end, 50 cycles sequencing on HiSeq2000 (Illumina). The abundance of mRNA and preferential mRNA regions from the CLIP assays are shown in Fig C in S1 File.

### High-throughput sequencing of RNA isolated by cross-linking immunoprecipitation (HITS-CLIP)

The Ago2 HITS-CLIP assay was carried out as described for PAR-CLIP unless indicated otherwise. The MDMs were cross-linked once with 150 mJ/cm^2^ at 254 nm on ice before lysis in NP40 and pooling of three different donors for HIV-1_JR-FL_ infected and non-infected samples.

### Small RNA sequencing

Total RNA was isolated from MDMs with TRIZOL according to the manufacturer’s protocol. 1μg of total RNA was processed for small RNA sequencing. Additionally, four calibrator oligonucleotides (Cal 01–04 5 fMol each) were added as a reference as described previously [37,38]. Briefly, RNA was dephosphorylated using FastAP (Fermentas) and radiolabelled as described above. Subsequently, RNA was separated with denaturing PAA (15%) gelelectrophoresis and fractions between 18 and 30 nt in size of were excised from the gel (Fig D in S1 File). Further steps were carried out in analogy to the PAR-CLIP protocol as described above.

### Accession codes

Deep sequencing data from PAR-CLIP, HITS-CLIP and small RNA sequencing has been deposited in NCBI’s Gene Expression Omnibus under GEO Series accession number GSE70851.

### Quantification of miRNAs and HIV-1 sncRNAs in macrophages

The quantification of cellular miRNAs and HIV-1 sncRNAs in macrophages was described previously [28]. Briefly, low molecular weight RNA (<200 nt) was isolated, 3’ C-tailed and reverse transcribed (M-MuLV Reverse Transcriptase, Finnzymes) with a C-tail specific linker primer (mf331 5’-ACCAGAGTGCGAGTAGGAAGATTGGGGGGGGG-3’). The miRNAs and previously described HIV-1 sncRNAs [28] were quantified by qPCR and normalized to miR-223 (Fig E in S1 File).

### Small RNA sequencing data analysis

Adapter sequences were removed from sequencing reads using the clipper tool from the FASTX toolkit [39]. Clipped reads shorter than 13 nucleotides were discarded. Remaining clipped reads were competitively aligned to the Ensembl human genome reference CRCh37, the HIV-1_JR-FL_ genome, and calibrator sequences using the Bowtie short read aligner [40]. The aligner was configured to allow one mismatch per read and to report a maximum of one hit per input read by randomly assigning ambiguous reads to one of the genomic regions they aligned to (bowtie parameters:–*l 50 –n 1 –e 30 –m 10 –k 1—best—strata—nomaqround*). Aligned reads were annotated to entries in Ensembl annotation for CRCh37.72, and the mature microRNAs in mirBase (Release 20) using HTSeq-count tool [41] Identical mature microRNAs derived from different chromosomal loci were pooled together. Differential expression tests were carried out using the Bioconductor package DESeq2 [42]. Samples were analyzed by paired tests in order to account for batch effects. Data normalization and dispersion estimation were performed using all expressed loci, while only miRNAs expressed above 10 reads were included in the final tests for differential expression in order to maintain statistical power. P-values were corrected to control the false discovery rate (FDR) using the Benjamini-Hochberg procedure [43], and those below 5% FDR were reported as significant.

### PAR-CLIP and HITS-CLIP data analysis

PAR-CLIP and HITS-CLIP samples were clipped and aligned the same. Adapters were removed from PAR-CLIP reads as described above for small RNA data. Reads were analyzed using PARalyzer [44]. The analysis pipeline was run with the default settings and the alignment was performed using the recommended parameters of the package (bowtie parameters:-v 2-m 10—best—strata). Only loci expressed by more than five reads were considered in the analysis. Potential miRNA target clusters were identified using PARalyzer for the mature miRNA sequences available in mirBase (Release 20).

### Expected read counts of virus-derived sncRNAs in PAR-CLIP assay

The relative expression of miRNAs in small RNA sequencing and PAR-CLIP was estimated using miRNAs expressed in both. The data was log-transformed and used in a linear regression model with zero intercept to estimate the expression ratio in paired samples. Confidence intervals for the parameter estimates and predicted PAR-CLIP counts were calculated using 10,000 bootstrap samples.

## Results

### HIV-1 sncRNAs and mRNAs in HIV-1_JR-FL_ infected MDMs were not incorporated in Ago2-RISC

To investigate whether HIV-1 derived sncRNAs are involved in post-transcriptional gene regulation mediated by endogenous Ago2, we applied PAR-CLIP in MDMs from two donors (donor 2 and donor 4) infected with HIV-1_JR-FL_ for 14 days and non-infected MDMs. Ago2 PAR-CLIP enables the identification of Ago2-bound miRNAs as well as miRNA-targeted RNAs with single-nucleotide resolution through diagnostic T-to-C mutations [35,36]. High-throughput sequencing of the Ago2-bound RNAs and followed by a competitive alignment strategy resulted in more than 26 million reads in a total of four libraries (Table 1), of which 2,146 reads (0.0081%) were aligned to the HIV-1 genome (Table 1). Six loci on the HIV genome were covered with at least five reads each, representing 1,847 reads in total, of which only the two groups in antisense orientation were identified to harbor T-to-C mutations (Table 2). The first cluster was abundantly present in all the samples including the non-infected MDMs. This region in the viral genome is the tRNA^Lys^ primer binding site, suggesting that at least a part of the respective tRNA might be incorporated into the Ago2 RISC. The second cluster with diagnostic T-to-C mutations mapped to Gag and had a low coverage of 12 reads in total, 11 of those showed identical T-to-C mutations derived from a single sample (Fig F in S1 File).

**Table 1:** High-throughput sequencing of RNA libraries derived by Ago2 PAR-CLIP and HITS-CLIP of HIV-1_JR-FL_ infected and non-infected monocyte-derived macrophages.

	Donor ID	HIV-1_JR-FL_	Total reads^a^	Reads aligned^b^	Reads aligned in %^b^	Reads aligned to HIV-1_JR-FL_ ^b^	Reads aligned to HIV-1_JR-FL_ in %^b^
**Ago2 PAR-CLIP**	Donor 2	+	12,470,608	5,846,963	46.89	143	0.0024
	Donor 4	+	16,822,014	4,289,309	25.5	311	0.0073
	Donor 2	-	13,595,536	6,814,986	50.13	294	0.0043
	Donor 4	-	31,073,335	9,433,653	30.36	1,398	0.0148
	Sum		73,961,493	26,384,911	35.67	2,146	0.0081
**Ago2 HITS-CLIP**	3 Donor Mix	+	24,497,870	6,807,427	27.79	584	0.0086
	3 Donor Mix	-	37,675,556	18,869,317	50.08	696	0.0037
	Sum		62,173,426	25,676,744	41.3	1,280	0.005

^a^Total reads represent reads after size selection (≥13 nts) and removal of adaptor-adaptor sequences

^b^Reads were competitively aligned to the human and the HIV-1_JR-FL_ genome. Read number aligned to either both genomes or HIV-1_JR-FL_ only are shown (Reads aligned) in addition to % of all reads (Reads aligned in %, Reads aligned to HIV-1_JR-FL_ in %).

**Table 2:** Characteristics of reads aligned to the HIV-1_JR-FL_ genome identified by AGO-2 PAR-CLIP in HIV-1_JR-FL_ infected (n = 2) and non-infected (n = 2) samples.

Strand	Start position^a^	End position^a^	Coverage^b^	T to C count^c^	Protein binding^d^
**Sense**	1,614	1,636	18	0	-
**Sense**	5,325	5,348	6	0	-
**Sense**	7,999	8,018	8	0	-
**Sense**	8,025	8,072	23	0	-
**Antisense**	637	655	1781	28	+
**Antisense**	801	817	11	11	+

^a^The location of each cluster mapping to the HIV-1_JR-FL_ genome is specified by its start and end position according to the HIV-1_HXB2_ reference genome (GenBank accession number K03455)

^b^The coverage shows the total number of reads aligned to loci

^c^T-to C counts are the numbers of observed T-to C mutations in the aligned reads

^d^Analysis of protein-binding (indicated with “+”) by PARAlyzer [44] analysis pipeline

To further exclude the possibility that viral RNAs were missed due the absence of Ts in the correct position and thus their inability of being cross-linked adequately, a similar but T-to-C conversion independent technique (HITS-CLIP) was applied. HITS-CLIP was performed in MDM in an additional three donor mix infected with HIV-1_JR-FL_ for 14 days and non-infected MDMs. The results revealed the absence of substantial amount of HIV-1 sncRNAs except some sncRNAs however similarly detected in HIV-1 infected and non-infected sample (Table 1 and S1 Table). Taken together, there was no evidence for HIV-1 sncRNA or mRNA incorporation in the Ago2- RISC

### Small RNA sequencing showed the presence of HIV-1 sncRNAs in HIV-1_JR-FL_ infected MDM

**Fig 1:**
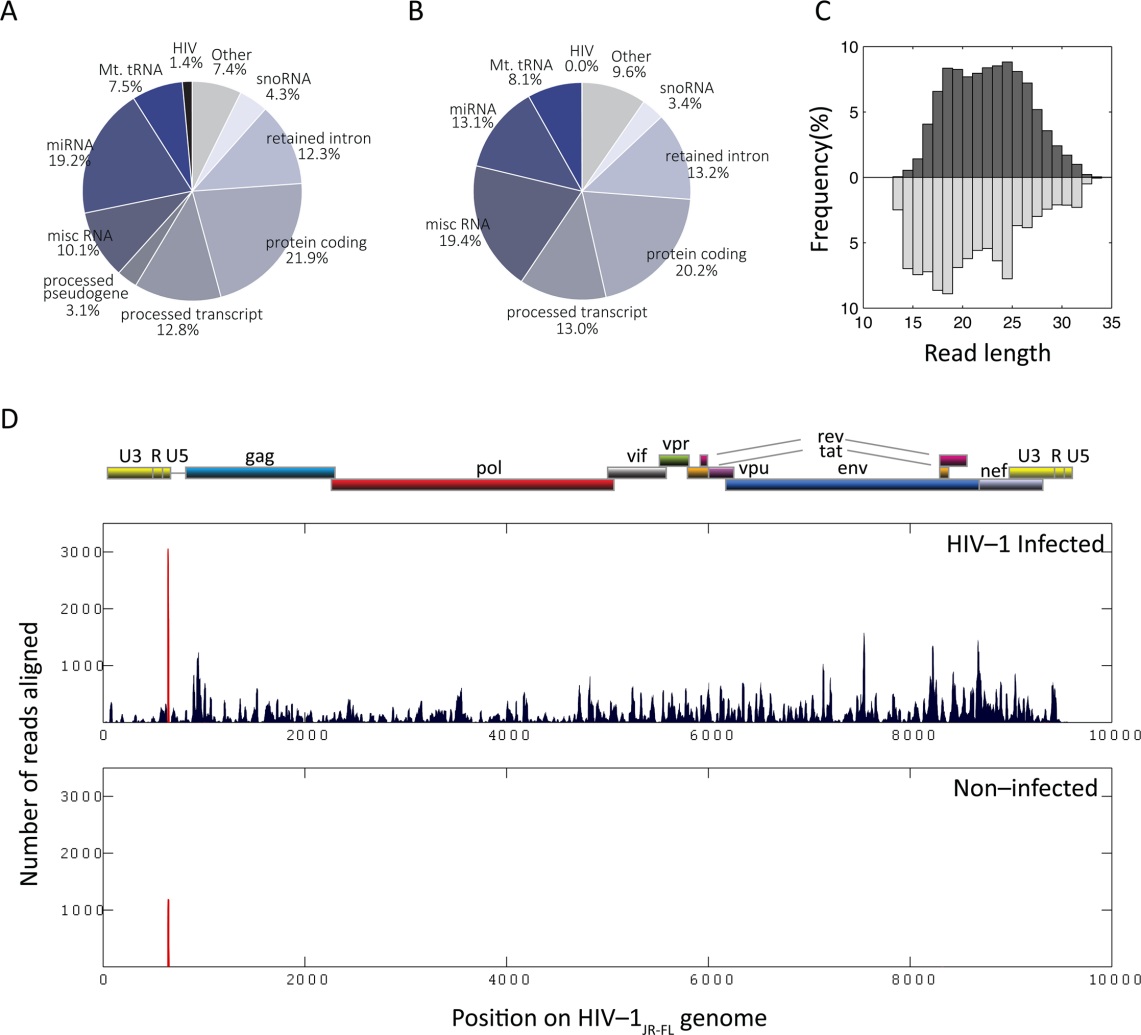
Characteristics of small RNAs derived from small RNA sequencing. Small RNA species detected in HIV-1_JR-FL_ infected MDMs (n = 4) (**A**), and non-infected MDMs (n = 4) (**B**). (**C**) Read length distribution of HIV-1 sncRNAs (dark grey) and total small RNA (light grey). (**D**) Small RNA sequencing reads (n = 4) aligned to the HIV-1_JR-FL_ genome. Transcripts aligned in antisense orientation are shown in red, predominantly representing tRNA^Lys^. Upper panel shows a diagram of the HIV-1 genome organization.

The absence of HIV-1 sncRNAs in the Ago2 PAR-CLIP and HITS-CLIP data prompted us to generate small RNA libraries from the MDM donor samples also used for the Ago2 PAR-CLIP. In addition, MDMs infected with HIV-1_JR-FL_ from two additional donors were analyzed. Over 65 million and 85 million reads from HIV-1_JR-FL_ infected (n = 4) and non-infected (n = 4) samples, respectively, were aligned to either the human or the HIV-1_JR-FL_ genome by competitive alignment strategy (Table 3). Sequencing of the total small RNA fraction yielded 19.2% and 13.1% miRNAs, and 21.9% and 20.2% protein-coding transcripts in the infected and non-infected samples, respectively (Fig 1A and 1B). The size distribution of HIV-1 sncRNA ranged from 16 to 29 nucleotides (corresponding to ~2.5–97.5% quantiles), reaching a plateau between 17 and 26 nucleotides, showing a distinct pattern compared to other total small RNA species (Fig 1C). HIV-1 sncRNAs comprised 1.4% of all small RNAs in HIV-1_JR-FL_ infected MDMs (n = 4) whereas they were represented only 0.01% in non-infected MDMs mainly represented by the host-derived tRNA_Lys_ (Fig 1D). HIV-1 sncRNAs were aligned to all regions of the HIV-1_JR-FL_ genome with hot spots in *gag* and *env* (Fig 1D). The majority of HIV-1 sncRNAs were derived from sense orientation (>99%), and the minority of antisense orientation was largely represented by tRNA_Lys_ present in both infected and non-infected samples (Fig 1D). The presence of HIV-1 sncRNAs, sncRNA in LTR and an antisence sncRNA in env [28], with 3’OH ends was further confirmed by qPCR in HIV-1_JR-FL_ infected samples (Fig D in S1 File). Both HIV-1 sncRNAs were present in all HIV-1_JR-FL_ infected samples and were absent in controls. The cellular miRNAs, miR-21 and miR-223 were highly abundant miRNAs in macrophages (qPCR data not shown). The levels of HIV-1 sncRNAs were comparable with cellular miRNAs expressed at lower levels [45,46].

**Table 3:** High-throughput sequencing of small RNA libraries of HIV-1_JR-FL_ infected (n = 4) and non-infected (n = 4) monocyte-derived macrophages.

	HIV-1_JR-FL_	Total reads^a^	Reads aligned^b^	Reads aligned in %^b^	Reads aligned to HIV-1_JR-FL_ ^b^	Reads aligned to HIV-1_JR-FL_ in %^b^
**Donor 1**	+	21,255,845	17,163,001	80.74	30,847	0.1797
**Donor 2**	+	17,913,668	14,335,116	80.02	3,431	0.0239
**Donor 3**	+	27,440,998	20,601,911	75.08	17,209	0.0835
**Donor 4**	+	16,347,062	12,988,200	79.45	22,330	0.1719
**Sum**	+	82,957,573	65,088,228		73,817	
**Donor 1**	-	17,635,535	14,547,394	82.49	101	0.0007
**Donor 2**	-	27,114,541	22,029,614	81.25	329	0.0015
**Donor 3**	-	21,175,108	17,481,336	82.56	196	0.0011
**Donor 4**	-	37,839,512	31,678,367	83.72	698	0.0022
**Sum**	-	103,764,696	85,736,711		1,324	

^a^Total reads represent reads after size selection (≥13 nts) and removal of adaptor-adaptor sequences

^b^Reads were competitively aligned to the human and the HIV-1_JR-FL_ genome. Read number aligned to either both genomes or HIV-1_JR-FL_ only are shown (Reads aligned) in addition to % of all reads (Reads aligned in %, Reads aligned to HIV-1_JR-FL_ in %).

### High concordance of small RNA sequencing and Ago2 PAR-CLIP in quantification of cellular miRNAs

**Fig 2:**
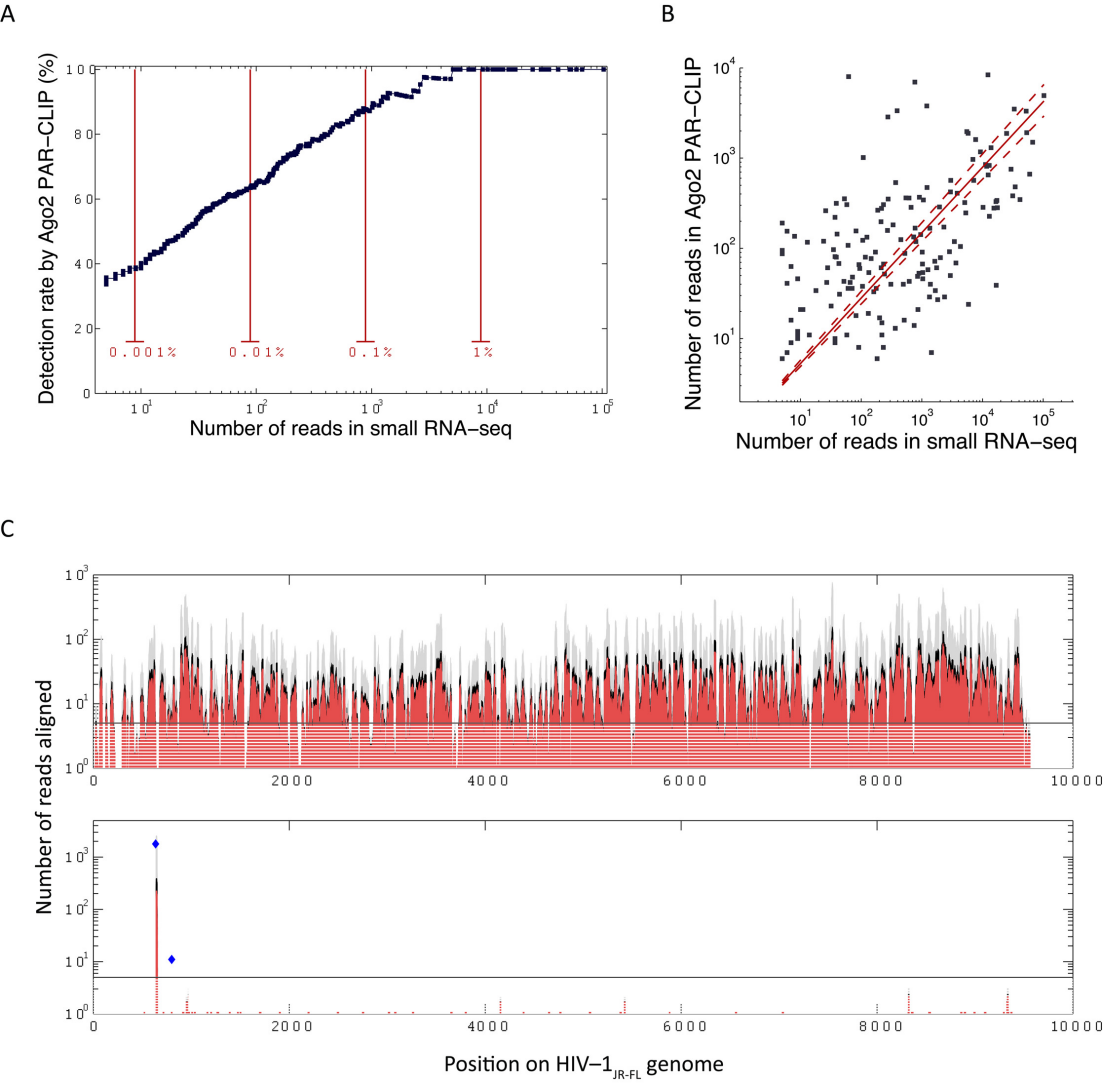
Cellular micro RNAs as detected by Ago2 PAR-CLIP and small RNA sequencing. **(A)** Percentage of the cellular miRNAs from small RNA sequencing that was also found in Ago2 PAR-CLIP as a function of minimum expression threshold. The x-axis shows the total abundance of miRNAs in small RNA sequencing data derived from pooled HIV-1_JR-FL_ infected (n = 4) and HIV-1 non-infected (n = 4) samples from the same donors. Red lines show the read count corresponding to specific fractions of total miRNA pool **(B)** Expression of cellular miRNAs in Ago2 PAR-CLIP and small RNA sequencing data is well correlated for 160 miRNAs found by both methods (*R* = 0.55, *p*<10^‒13^). The dashed lines show bootstrap-derived 95% confidence intervals for the linear fit (red line). **(C)** Expected PAR-CLIP read counts of virus-derived sncRNAs associated with Ago2. Adjusted to small RNA sequencing data derived from pooled HIV-1_JR-FL_ infected (n = 2) samples matching the PAR-CLIP donors. The black line (5 reads aligned) depicts the detection limit of the PAR-CLIP assay with majority of the loci on the sense genome are expected to surpass the detection limit. Upper and lower panels correspond to the reads on the sense and anti-sense strand of HIV-1_JR-FL_ genome respectively.

Although highly abundant in small RNA sequencing, HIV-1 sncRNAs were almost completely absent in the Ago2 PAR-CLIP libraries. In order to evaluate the quality of our PAR-CLIP assays, we focused our analysis on the known miRNAs, assuming that the majority of expressed miRNAs can be associated with Ago2-RISC and hence should be present in Ago2 PAR-CLIP data. Pooled Ago2 PAR-CLIP and small RNA sequencing libraries captured 183 and 475 unique mature miRNAs that were present by at least five reads. 160 miRNAs were detected by both assays. The relative sensitivity of the Ago2 PAR-CLIP assay was assessed as a function of miRNA abundance in the small RNA sequencing experiment. We observed a strong correspondence between miRNA frequency in total miRNA library (n = 4) and its detection by Ago2 PAR-CLIP (n = 4) (Fig 2A). 88% of all cellular miRNAs—detected with ≥1,000 reads in small RNA sequencing—were also observed in PAR-CLIP (Fig 2A). This rate dropped only to 64% and 38% when ≥100 and ≥10 reads, respectively, were chosen as cut-offs. Even more than 30% of low abundant miRNAs, i.e., detected with only ≥5 reads, were identified in the PAR-CLIP data (Fig 2A). Furthermore, the expression level of the miRNAs was highly correlated between the two methods for the 160 miRNAs detected in both datasets (*R* = 0.55, *p*<10^‒13^) (Fig 2B). Given the strong concordance of the two methods in detecting cellular miRNAs we expected potential viral miRNAs or target sites—if associated with Ago2—to be also present in the Ago2 PAR-CLIP data. Fig 2C demonstrates HIV-1 reads observed in small RNA sequencing for the two PAR-CLIP matched donors, and indicates indicates the expected read count in our PAR-CLIP data for authentic Ago2 associated HIV-1 sncRNAs based on our observations using cellular miRNA data (Fig 2A and 2B). Despite the detection of four highly abundant HIV-1 sncRNAs (>500 reads) and 107 abundant HIV-1 sncRNAs (>100 reads) by small RNA sequencing none of them were present in the PAR-CLIP data (Fig 2C). Only one HIV-1 sncRNA was observed in both, the small RNA sequencing and the PAR-CLIP data (Fig 2C), but this HIV-1 sncRNA corresponded to the tRNA^Lys^ primer binding site and was also detected in HIV-1 non-infected samples (Fig F in S1 File). Moreover, the highly abundant reads on the sense strand of the virus, equivalent to 9.1% of miRNA pool, in the small RNA sequencing data was entirely absent in the PAR-CLIP and hence it is highly unlikely that HIV-1 sncRNAs were missed due to the detection limit of the PAR-CLIP assay.

## Discussion

Several studies have identified HIV-1 encoded sncRNAs in different experimental setups, several of those have been suggested to function as virally encoded miRNAs [12,13,21,22,26–28]. However, two earlier studies failed to identify HIV encoded miRNAs by using conventional sequencing of HIV-1 infected HeLa cells and ACH2 T cells [7,30]. Another more recent study showed no evidence for the existence of HIV encoded miRNAs by applying Ago PAR-CLIP assays in an HIV-1 infected T cell line (C8166) and TZM-bl epithelial cells [29].

The present study aimed to characterize the role of HIV-1 sncRNAs in the context of RNAi pathway in human primary macrophages, an important HIV-1 reservoir by specifically addressing two questions. First, do HIV-1 sncRNAs emulate canonical miRNAs and associate with RISC to regulate HIV-1 or host gene expression? Second, do HIV-1 transcripts present as targets for the host miRNA pathway?

To address these questions, Ago2 PAR-CLIP was applied on HIV-1_JR-FL_ infected and non-infected MDMs from two different donors. Competitive alignment strategy to the human and viral genome was applied to avoid false positive hits on the viral genome. Less than 0.01% of reads were aligned to HIV-1. They formed six clusters, only two of the loci harbored the indicative T-to-C mutations that are required for excluding background sequences not incorporated in Ago2-RISC. One was identified as tRNA^Lys^, the HIV-1 reverse transcription primer, suggesting that tRNA^Lys^ may be recognized and associated with RISC. This finding is in line with previous reports of Ago2 associated tRNA^Lys^ in HIV-1 infected cells [26], and the miRNA-like molecules derived from tRNAs observed in various studies [26,47–51], but in contrast to the work from Whisnant and colleagues who did not detect tRNALys being associated with Ago2. The results might be explained with different read processing, alignment strategies or the use of other cell sources for the generation of PAR-CLIP data [29]. The second HIV-1 sncRNA, aligned at nucleotide position 801 to HIV-1_JR-FL_, detected by Ago2 PAR-CLIP, and most likely does not represent a virus-encoded miRNA or a target sequence due to its sequence count, length and distribution. The Ago2 HITS-CLIP data from a three donor mix of HIV-1 infected and non-infected MDM (S1 Table) confirmed the absence of HIV-1 sncRNAs in RISC. **The presence of tRNA** ^**Lys**^ **in both, HITS-CLIP and PAR-CLIP data, suggests the potential of being associated with Ago2-RISC, however, the fact that they were identified in infected and non-infected cell lysates excludes HIV-1 specificity.**

The expression of HIV-1 sncRNAs in infected MDMs was confirmed with small RNA sequencing on cell lysates. Over 150 million reads were obtained after simultaneous and competitive alignment to the human and HIV-1_JR-FL_ genome indicating good library diversity and depth. HIV-1 sncRNAs represented about 1.22% of the whole small RNA fraction and were almost absent (0.01%) in non-infected controls. The percentage of identified HIV-1 reads in the small RNA fraction is in line with previous observations, as well as distribution of HIV-1 sncRNAs along the viral genome with several hotspots shared between all donors [26–29]. Moreover, read clusters from our previous dataset of HIV-1_JR-FL_ infected macrophages, collected by hybridization capture probe enrichment based methodology, correlated well with our current data set [28], confirming the absence of technology-dependent biases and artifacts. HIV-1 transcripts were entirely absent in Ago2 PAR-CLIP in sharp contrast to the general concordance of small RNA sequencing and Ago2 PAR-CLIP in identifying cellular miRNAs. Taken together, this suggests that HIV-1 neither expresses canonical miRNAs nor displays potential targets for miRNA-Ago2 guided degradation. One could argue that the sensitivity of Ago2 PAR-CLIP was not high enough to identify HIV-1 encoded miRNAs. Indeed, the Ago2 PAR-CLIP assays were performed on low cell amount, ranging from 30 to 60 million MDM and representing only 1/50 of cell input amount used by other groups [35]. However, this is less likely, since highly abundant miRNAs were found in both, PAR-CLIP and small RNA sequencing. In fact, cellular miRNAs expressed above 10, 100, and 1,000 reads in small RNA sequencing were also present in PAR-CLIP in over 38%, 64%, and 88% of the times, respectively, and 100% for miRNAs detected with more than 5,000 reads. Therefore, the simultaneous absence of almost all HIV-1 sncRNA reads detected by small RNA sequencing in PAR-CLIP data cannot be explained by the detection limit of the assay. In case of efficient HIV-1 targeting by host miRNAs we would expect enrichment of HIV-1 reads in the PAR-CLIP data. Indeed, our bioinformatic data analysis deduced from small RNA sequencing and Ago2 PAR-CLIP high concordance between the two data sets (R = 0.55, p<10^‒13^). The highly abundant as well as miRNAs with 0.5% of the total miRNA pool were present in the PAR-CLIP data. These results suggest that we would not have missed virally encoded sncRNAs in Ago2 PAR-CLIP if they would have been present.

It cannot be ruled out entirely that the HIV-1 sncRNAs are–at least partly–degradation products. However, the classical pathways of RNA degradation generate 3’ends which are not suitable for our C-tailing procedures applied for the quantification of the HIV-1 sncRNAs [52]. In addition, the low proportion of cellular RNA degradation products in our sequence reads does also not allow the conclusion that mainly RNA degradation products were selected. The absence of HIV-1 transcripts among the targets of host miRNA pathway may be explained by the highly conserved secondary structures of HIV-1 RNA [53], which can protect the virus from the cellular RNA interference pathway [54]. Other studies [55,56] showed an association of RISC proteins and HIV-1 RNA, however, these immune precipitation experiments do not confirm direct incorporation of HIV-1 RNA into the binding pocket of Ago2. PAR-CLIP conditions allow protein protected enrichment of RNA fragments due to RNAse digestion prior to Ago2-IP [34]. Whisnant *et al*. investigated Ago protected HIV-1 RNAs [29]. In line with this study we do not observe any authentic HIV-1 sncRNAs acting in a mi/siRNA like manner, as well as we did not observe HIV-1 being targeted by cellular miRNAs since HIV-1 reads were almost absent in our PAR-CLIP data.

## Conclusions

Our data show that HIV-1 sncRNAs in HIV-1_JR-FL_ infected MDMs are not incorporated as functional miRNAs, nor are they targets for host miRNAs associated with Ago2-RISC. The absence of endogenous HIV-1 sncRNA and mRNA from Ago2-RISC suggests that HIV-1 has developed mechanisms to circumnavigate the canonical RNAi pathway and thus, may have evolved the ability to outsmart the host by counteracting this defense strategy.

## Supporting Information

S1 File Fig A—PAR-CLIP Ago2-autoradiogram of donor 4 for HIV-1_JR-FL_ infected and non-infected macrophages. Ago2 was cross-linked and immunoprecipitated with Ago2-antibody coated ProtG magnetic beads. The Ago2 bound RNA was dephosphorylated and ‘radiolabeled with ATP ([γ-^32^P]. The protein-RNA complexes were separated by SDS-PAGE. The box represents Ago2 and was cut from the gel. **Fig B—Efficiency of Ago2-immuno precipitation.** Cellular miRNAs miR-23a and miR-21 were measured after Ago2-IP by qPCR in macrophages of three donors and shown as fold enrichment compared to 5S rRNA. **Fig C—mRNA regional preference in CLIP datasets.** The current CLIP dataset was applied to CLIPz [60] and the presence of mRNA and mRNA regional preference was analyzed. The abundance of mRNA in the PAR CLIP data is ~3.5% and thus comparable to other datasets [35,36]. The majority of the mRNA was found to derive from 3’UTR, which is known to contain the major target binding sites for cellular miRNAs. **Fig D—Small RNA sequencing sample preparation.** Total RNA was isolated from macrophages of four donors infected with HIV-1_JR-FL_. 1 μg of total RNA was separated by denaturing PAA (15%) gelelectrophoresis and the size fraction between 18 to 30 nt were excised from gel. **Fig E–Quantification of mi/sncRNAs by qPCR in HIV-1**_**JR-FL**_ **infected (n = 3) and non-infected (n = 3) primary macrophages.** Cellular miRNAs miR-223, miR-21 (dark grey) and known HIV-1_JR-FL_ derived small RNAs from two different contigs [28], namely sncRNA in LTR (orange) and env (pink), were quantified. The Ct values were normalized to miR-223. HIV-1 sncRNAs were detected in HIV-1 infected samples (grey shaded area) in biologically relevant expression levels. **Fig F—HIV-1 associated PAR-CLIP clusters.** Two virus-associated clusters were found carrying the indicative T-to-C mutations, both in antisense orientation. The left and right panels show the read coverage patterns of the first (primer binding site) and the second cluster in *gag*, respectively, in each sample. The T-to-C mutations are shown in black (A-to-G on antisense strand), other mismatches are shown in dark grey. The position on the HIV_JR-FL_ genome is shown on the top, and the reference viral genome sequence is shown on the bottom axis.
(PDF)

S1 Table Characteristics of reads aligned to the HIV-1_JR-FL_ genome identified by AGO2 HITS-CLIP in HIV-1_JR-FL_ infected (3 donor mix) and non-infected (3 donor mix) samples. The location of each cluster mapping to the HIV-1_JR-FL_ genome is specified by its start and end position according to the HIV-1_HXB2_ reference genome (GenBank accession number K03455) and the coverage shows the total number of reads aligned to the loci.
(XLSX)

S2 Table Small RNA sequencing raw data. Listed are the Gene ID, Gene Symbol, Type of RNA for Donor 1 to Donor 4 of HIV-1_JR-FL_ infected and non-infected.
(XLSX)
