# Supplementary material for: HIV-1 RNAs are Not Part of the Argonaute 2 Associated RNA Interference Pathway in Macrophages
Source: PLoS One. 2015 Jul 30;10(7):e0132127. doi: 10.1371/journal.pone.0132127 (PMC4520458; doi:10.1371/journal.pone.0132127)

Supporting Information – S1 File

Figure A - PAR-CLIP Ago2-autoradiogram of donor 4 for HIV-1$_{JR-FL}$ infected and non-infected macrophages. Ago2 was crosslinked and immunoprecipitated with Ago2-antibody coated ProtG magnetic beads. The Ago2 bound RNA was dephosphorylated and radiolabeled with ATP ([γ-^{32}P]. The protein-RNA complexes were separated by SDS-PAGE. The box represents Ago2 and was cut from the gel.

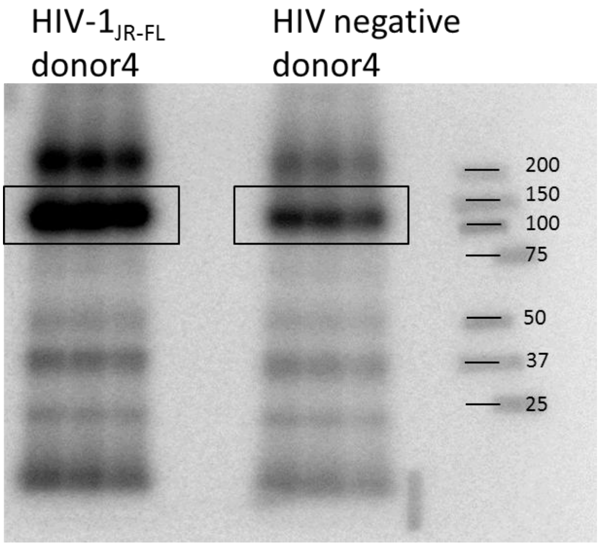

Figure B - Efficiency of Ago2-immuno precipitation. Cellular miRNAs miR-23a and miR-21 were measured after Ago2-IP by qPCR in macrophages of three donors and shown as fold enrichment compared to 5S rRNA.

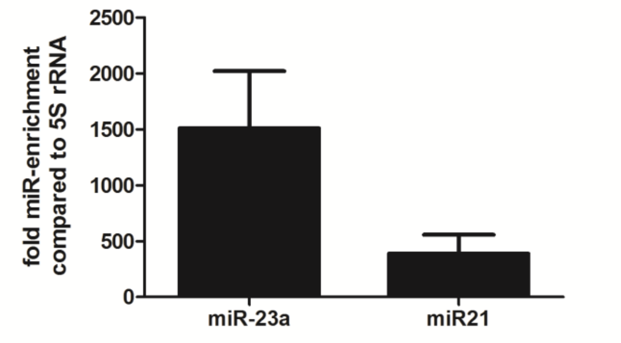

Figure C - mRNA regional preference in CLIP datasets. The current CLIP dataset was applied to CLIPz [60] and the presence of mRNA and mRNA regional preference was analyzed. The abundance of mRNA in the PAR CLIP data is ~3.5 % and thus comparable to other datasets [35,36]. The majority of the mRNA was found to derive from 3'UTR, which is known to contain the major target binding sites for cellular miRNAs.

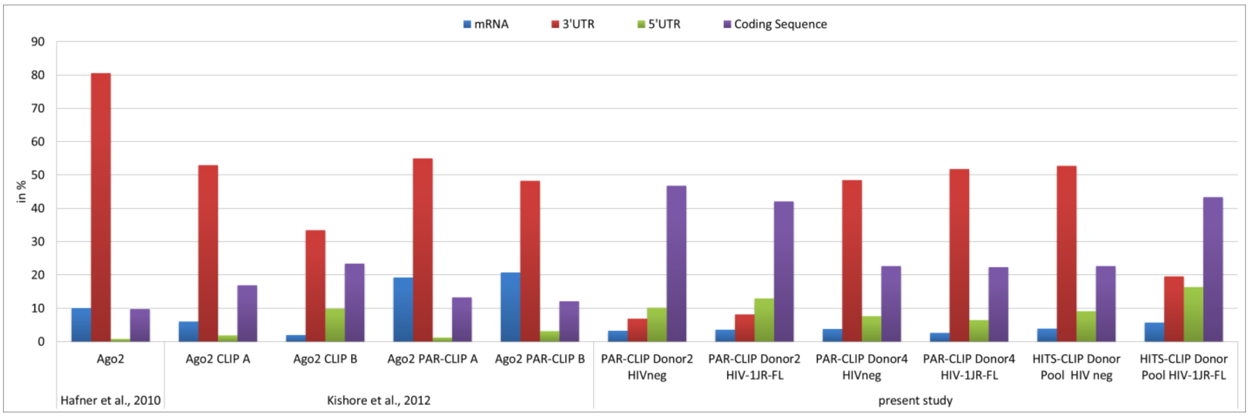

Figure D - Small RNA sequencing sample preparation. Total RNA was isolated from macrophages of four donors infected with HIV-1_{JR-FL}. 1 µg of total RNA was separated by denaturing PAA (15%) gelelectrophoresis and the size fraction between 18 to 30 nt were excised from gel.

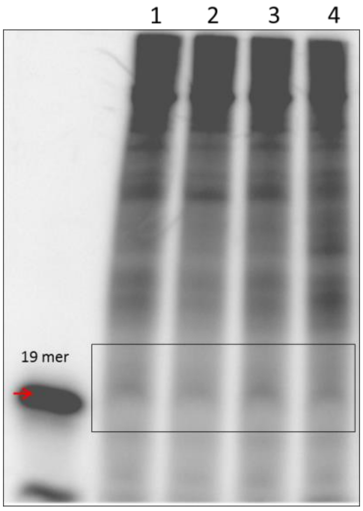

Figure E – Quantification of mi/sncRNAs by qPCR in HIV-1$_{JR-FL}$ infected (n=3) and non-infected (n=3) primary macrophages. Cellular miRNAs miR-223, miR-21 (dark grey) and known HIV-1$_{JR-FL}$ derived small RNAs from two different contigs [28] namely sncRNA in LTR (orange) and env (pink), were quantified. The Ct values were normalized to miR-223. HIV-1 sncRNAs were detected in HIV-1 infected samples (grey shaded area) in biologically relevant expression levels.

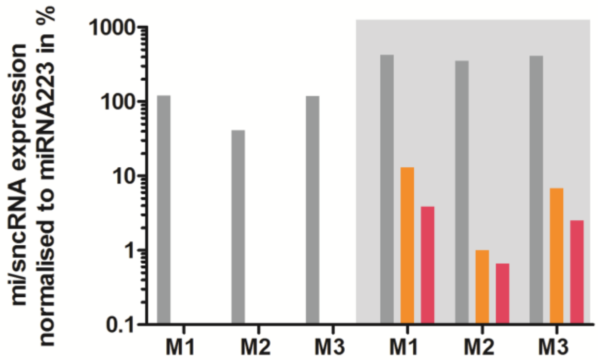

Figure F - HIV-1 associated PAR-CLIP clusters. Two virus-associated clusters were found carrying the indicative T-to-C mutations, both in antisense orientation. The left and right panels show the read coverage patterns of the first (primer binding site) and the second cluster in *gag*, respectively, in each sample. The T-to-C mutations are shown in black (A-to-G on antisense strand), other mismatches are shown in dark grey. The position on the $HIV_{JR\text{-}FL}$ genome is shown on the top, and the reference viral genome sequence is shown on the bottom axis.

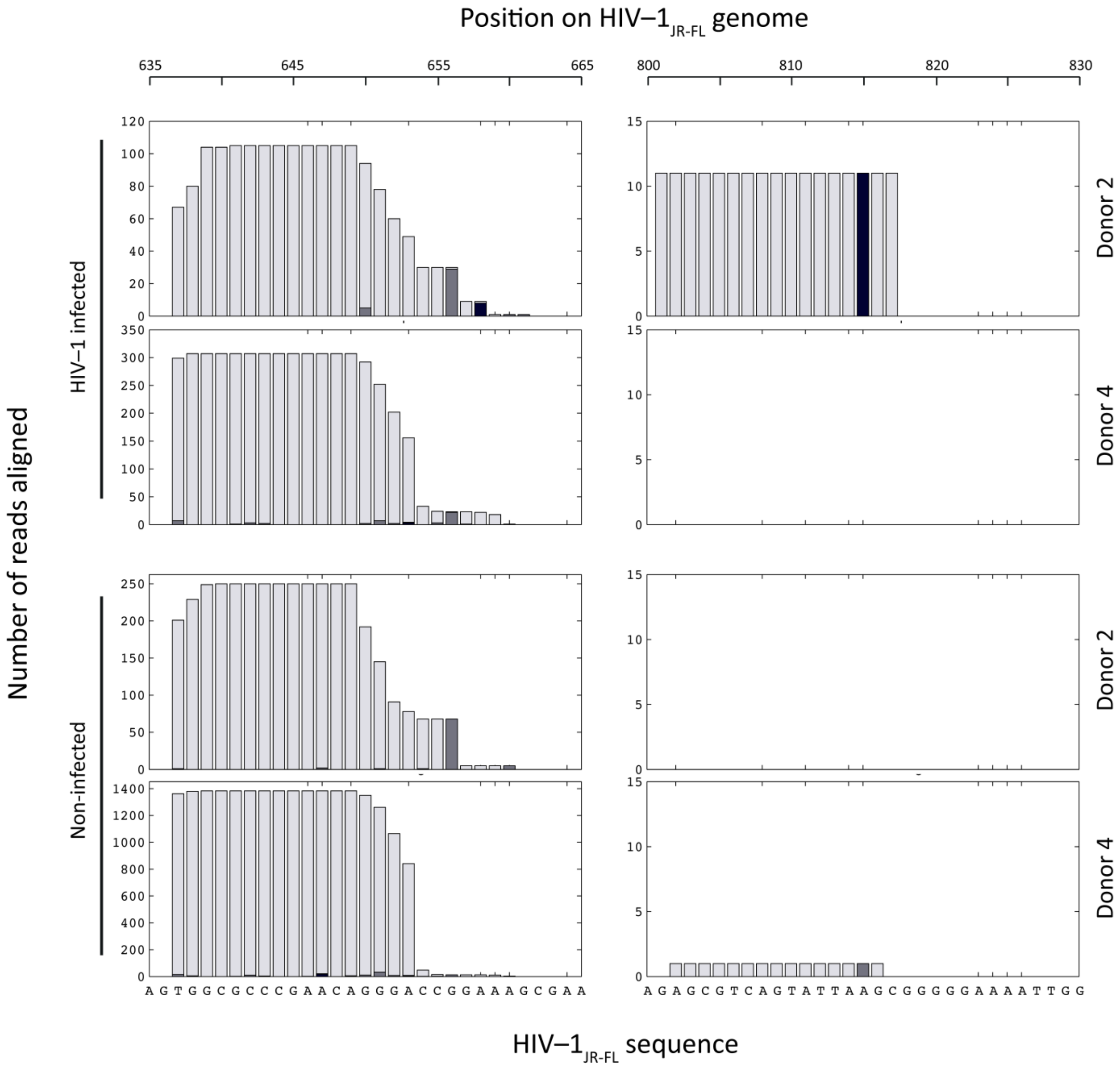

Supplement: S1 File — Ago2 was cross-linked and immunoprecipitated with Ago2-antibody coated ProtG magnetic beads. The Ago2 bound RNA was dephosphorylated and ‘radiolabeled with ATP ([γ-32P]. The protein-RNA complexes were separated by SDS-PAGE. The box represents Ago2 and was cut from the gel. Fig B—Efficiency of Ago2-immuno precipitation. Cellular miRNAs miR-23a and miR-21 were measured after Ago2-IP by qPCR in macrophages of three donors and shown as fold enrichment compared to 5S rRNA. Fig C—mRNA regional preference in CLIP datasets. The current CLIP dataset was applied to CLIPz [60] and the presence of mRNA and mRNA regional preference was analyzed. The abundance of mRNA in the PAR CLIP data is ~3.5% and thus comparable to other datasets [35,36]. The majority of the mRNA was found to derive from 3’UTR, which is known to contain the major target binding sites for cellular miRNAs. Fig D—Small RNA sequencing sample preparation. Total RNA was isolated from macrophages of four donors infected with HIV-1JR-FL. 1 μg of total RNA was separated by denaturing PAA (15%) gelelectrophoresis and the size fraction between 18 to 30 nt were excised from gel. Fig E–Quantification of mi/sncRNAs by qPCR in HIV-1 JR-FL infected (n = 3) and non-infected (n = 3) primary macrophages. Cellular miRNAs miR-223, miR-21 (dark grey) and known HIV-1JR-FL derived small RNAs from two different contigs [28], namely sncRNA in LTR (orange) and env (pink), were quantified. The Ct values were normalized to miR-223. HIV-1 sncRNAs were detected in HIV-1 infected samples (grey shaded area) in biologically relevant expression levels. Fig F—HIV-1 associated PAR-CLIP clusters. Two virus-associated clusters were found carrying the indicative T-to-C mutations, both in antisense orientation. The left and right panels show the read coverage patterns of the first (primer binding site) and the second cluster in gag, respectively, in each sample. The T-to-C mutations are shown in black (A-to-G on antisense strand), other mismatches [file pone.0132127.s001.pdf]
